# Supplementary material for: A non-genotoxic stem cell therapy boosts lymphopoiesis and averts age-related blood diseases in mice
Source: Nat Commun. 2025 Jun 2;16:5129. doi: 10.1038/s41467-025-60464-3 (PMC12130210; doi:10.1038/s41467-025-60464-3)
Supplement: Supplementary file 1 — Supplementary Information [file 41467_2025_60464_MOESM1_ESM.pdf]

Supplementary Data for

A Non-Genotoxic Stem Cell Therapy Boosts Lymphopoiesis and Averts Age-Related Blood Diseases in Mice

Anna Konturek-Ciesla[1,2], Qinyu Zhang[1], Shabnam Kharazi[1], David Bryder[1]*

Affiliations:

[1]Division of Molecular Hematology, Department of Laboratory Medicine, Lund Stem Cell Center, Medical Faculty, Lund University, Lund, Sweden.

[2]Department of Biosystems Science and Engineering, ETH Zurich, Basel, Switzerland.

*Corresponding author. Email: David.Bryder@med.lu.se

Contents:

Supplementary Figure 1

Supplementary Figure 2

Supplementary Figure 3

Supplementary Figure 4

Supplementary Figure 5

Supplementary Figure 6

Supplementary Table 1

Detailed information on transplantation experiments. Related to Fig. 1-5.

Supplementary Table 2

List of antibodies used in this study.

Supplementary Fig. 1

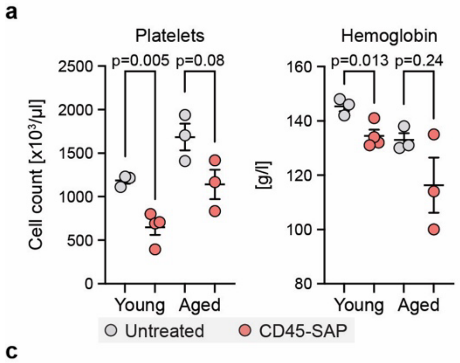

b

Cell count [x10³/µl]	Young Untreated	Young CD45-SAP		Aged Untreated	Aged CD45-SAP	
T cells	2.1± 0.2	0.6 ± 0.2	(p=0.002)	0.7± 0.3	0.3 ± 0.1	(p=0.17)
B cells	2.8 ± 0.4	1.0 ± 0.6	(p=0.004)	1.6 ± 0.5	0.9 ± 0.2	(p=0.12)
Myeloid cells	0.6 ± 0.2	2.6 ± 0.6	(p=0.004)	1.0 ± 0.3	3.5 ± 1.3	(p=0.06)
NK cells	0.2 ± 0.1	0.1 ± 0.02	(p=0.140)	0.1 ± 0.02	0.1 ± 0.03	(p=0.08)

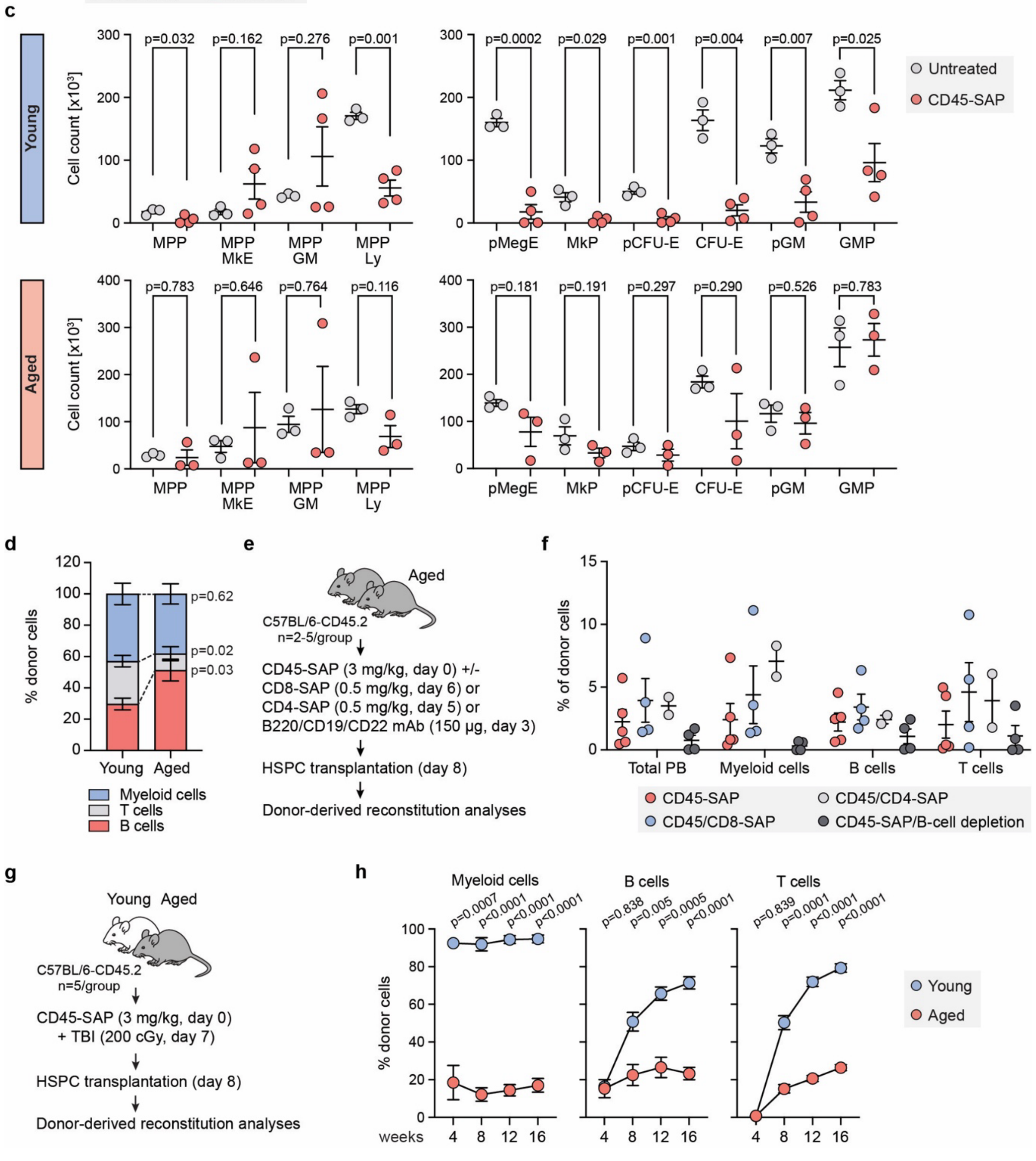

Supplementary Figure 1. The aged bone marrow environment restrains HSC engraftment (Related to Fig. 1).

(**a**) Young (2 months, n = 4) and aged (16 months, n = 3) C57BL/6-CD45.2 mice received CD45-SAP (3 mg/kg), and hematopoietic parameters were analyzed after 8 days. Untreated mice served as controls (n = 3 for both young and aged groups). Platelet counts (*left*) and hemoglobin levels (*right*) in control and CD45-SAP-treated mice. (**b**) Peripheral blood (PB) lymphoid and myeloid cell counts in control and CD45-SAP-treated mice. P-values are indicated in parentheses. Shown is the mean ± SD. (**c**) Absolute numbers of indicated hematopoietic stem and progenitor cell (HSPC) subsets in young (*top*) and aged (*bottom*) control and CD45-SAP-treated mice. In (a) and (c), grey and red circles represent untreated and CD45-SAP-treated groups, respectively. (**d**) Distribution of myeloid (blue), T cell (grey), and B cell (blue) lineages within donor-derived PB cells in young and aged recipients. (**e**) Experimental design for Extended Data Fig. 1e. Aged (16 months) C57BL/6-CD45.2 mice received CD45-SAP (3 mg/kg, n = 5) or CD45-SAP (3 mg/kg) with CD8-SAP (0.5 mg/kg, n = 4), CD4-SAP (0.5 mg/kg, n = 2) or B cell-depleting antibody cocktail (rat anti-mouse B220, anti-CD19 and anti-CD22, followed by anti-rat kappa light chain; 150 µg/mouse each antibody, n = 4). After treatment, mice were transplanted with HSPCs derived from young donor mice. (**f**) Donor-derived reconstitution in indicated PB lineages in aged recipients treated with CD45-SAP (red circle), CD45-SAP with CD8-SAP (blue circle), CD45-SAP with CD4-SAP (grey), or CD45-SAP with B cell-depleting antibody cocktail (dark grey circle) 16 weeks after transplantation. (**g**) Experimental design for Extended Data Fig. 1g. Young (2 months, n = 5) and aged (16 months, n =5) C57BL/6-CD45.2 mice received CD45-SAP (3 mg/kg) and low-dose total body irradiation (TBI, 200 cGy). Following treatment, mice were transplanted with HSPCs derived from young mice. (**h**) Donor-derived reconstitution in indicated PB lineages in young (blue circles) and aged (red circles) recipients. In (a), (b), (d), and (f), points indicate individual mice. Shown is mean ± SEM. Statistical significance was determined using an unpaired two-sided *t*-test with Welch correction. See also Supplementary Table 1. Source data are provided as a Source data file.

Supplementary Fig. 2

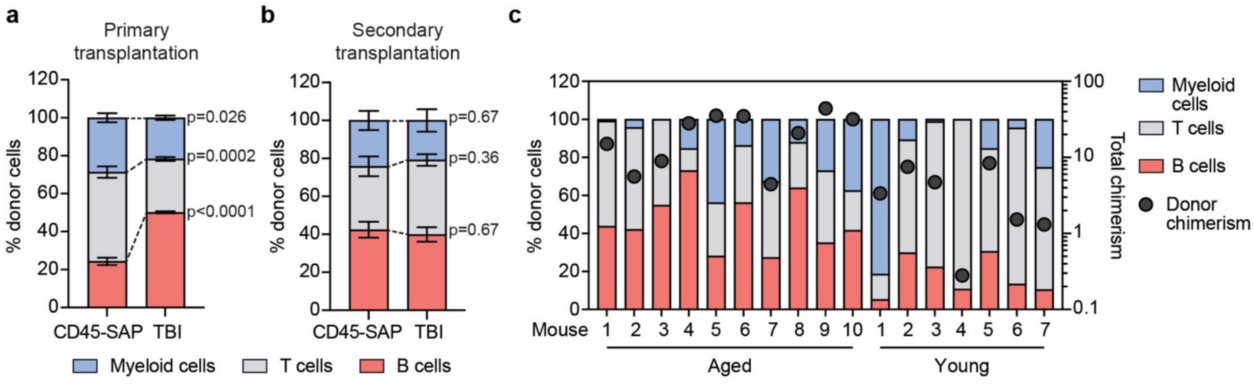

Supplementary Figure 2. Impact of host age and conditioning regimen on donor cell lineage distribution within peripheral blood. (Related to Fig. 2 and 3).

(a-b) Distribution of myeloid (blue), T cell (grey), and B cell (blue) lineages within donor-derived peripheral blood cells 18 weeks after transplantation in CD45-SAP- (n = 5) and TBI-treated (n = 5) primary (a) and secondary (b) recipients. (**c**) Distribution of myeloid (blue), T cell (grey), and B cell (blue) lineages and total donor-derived PB reconstitution (dark grey circle) levels 16 weeks after secondary transplantation into young, lethally irradiated recipients. The primary hosts were young and aged unconditioned mice. N = 7 for young and n = 10 for aged primary hosts. In (a) and (b), shown is mean ± SEM, and statistical significance was determined using an unpaired two-sided *t*-test with Welch correction. Source data are provided as a Source data file.

Supplementary Fig. 3

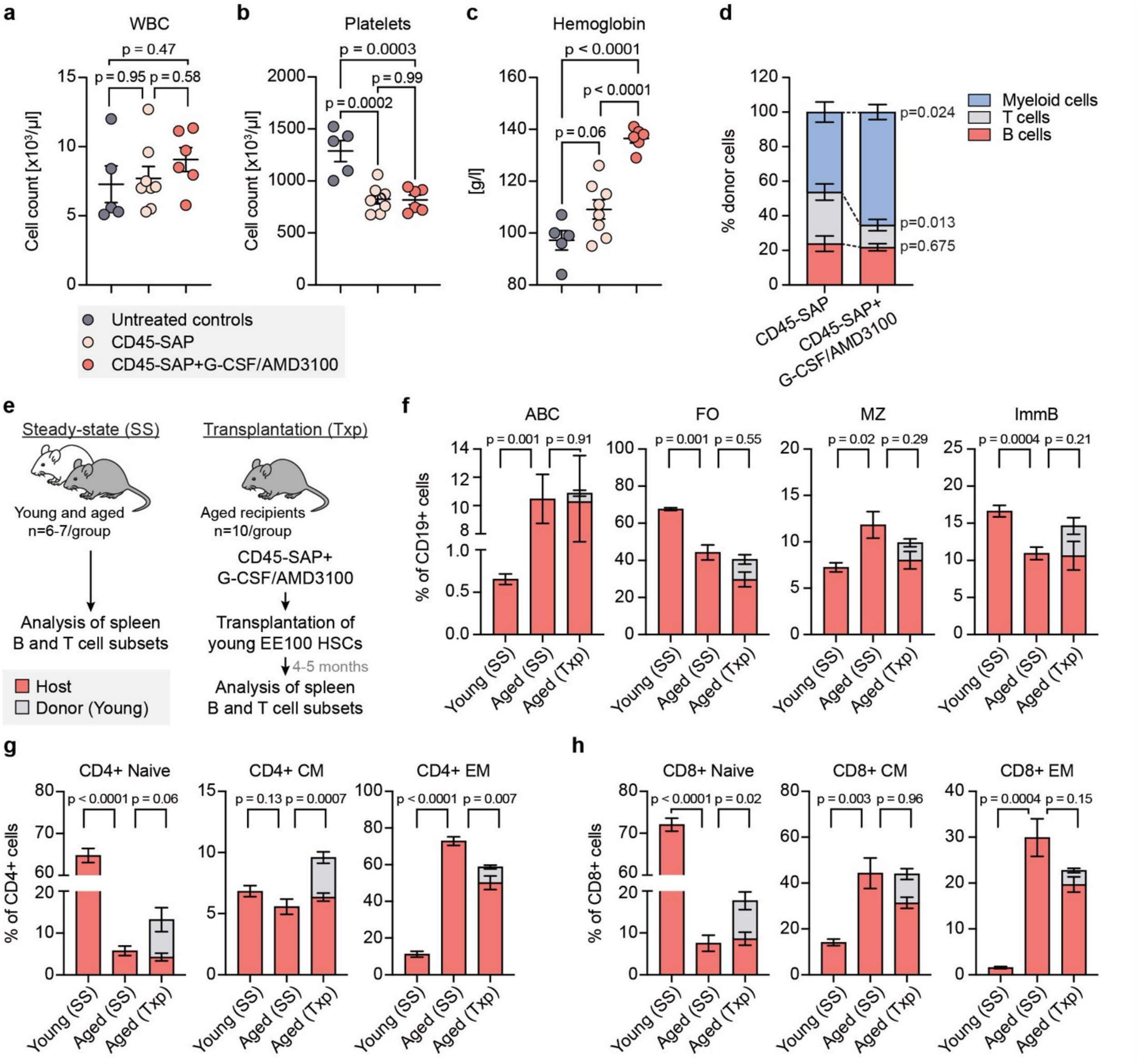

Supplementary Figure 3. Impact of CD45-SAP treatment on adaptive immune compartments in mice (Related to Fig. 4).

(**a-c**) White blood cell (a), platelet counts (b), and hemoglobin levels (c) in aged (16 months) recipients conditioned with CD45-SAP (n = 8, grey) or CD45-SAP with G-CSF/AMD3100 (n = 6, red). Untreated age-matched controls were assessed for comparison (n = 5, dark grey). (**d**) Distribution of myeloid (blue), T cell (grey), and B cell (blue) lineages within donor-derived peripheral blood cells 18 weeks after transplantation. (e) Experiment design for Supplementary Fig. 3f-h. Analysis of splenic B and T cell subsets was carried out in steady-state young (4-5

months, n = 6) and aged (22 months, n = 7) mice (*left*). The parallel comparison was performed in aged mice treated with CD45-SAP and G-CSF/AMD3100 and transplanted with *ex vivo* expanded young HSCs (EE100 cells/mouse, n = 10). (**f**) Frequency of age-associated B cells (ABC), marginal zone (MZ), follicular (FO), and immature (ImmB) B cells within splenic CD19+ cell fractions in aged steady-state and transplanted mice. (**g-h**) Frequency of naive, central memory (CM), and effector memory (EM) T cells within CD4+ and CD8+ splenic cell fractions in aged steady-state and transplanted mice. Shown is the mean ± SEM. In (f-h), grey and red bars denote donor and host groups, respectively. Statistical significance was determined using one-way ANOVA (a-c) and an unpaired two-sided *t*-test with Welch correction for two-group comparisons (d and f-h). Source data are provided as a Source data file. SS, steady state; Txp, transplantation.

Supplementary Fig. 4

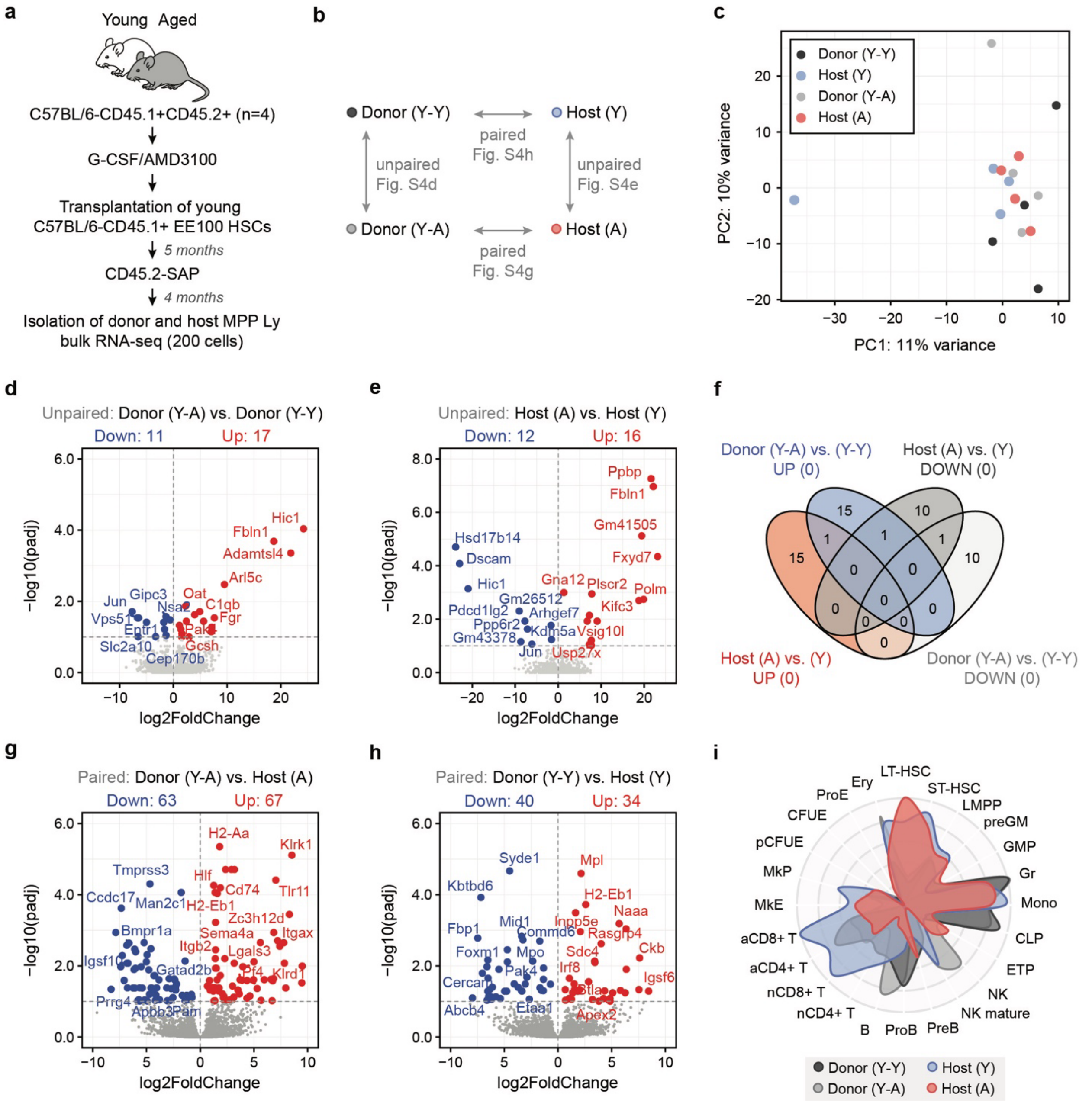

Supplementary Figure 4. Young MPP Ly cells retain their molecular profile in an aging bone marrow environment (Related to Fig. 4).

(a) Experiment design for Supplementary Fig. 4c-i. Young (2-3 months, n =4) and aged (16 months, n =4) C57BL/6-CD45.1/CD45.2 mice were conditioned with G-CSF/AMD3100 and transplanted with *ex vivo* expanded young HSCs (EE100 cells/mouse) derived from C57BL/6-CD45.1. Five months post-transplantation, the mice received a single dose of CD45.2-SAP (20

μg/mouse). Donor- and host-derived MPP Ly were isolated from the bone marrow four months after CD45-SAP treatment. A total of 200 cells per sample were sorted into TRIzol and subsequently used for cDNA library preparation and sequencing. (**b**) Overview of the analysis and sample comparisons. (**c**) Principal component analysis (PCA) of donor and host-derived MPP Ly cells isolated from young and aged transplantation recipients. The plot illustrates the contribution of each principal component to total variance. (**d-e**) Volcano plots of differentially expressed genes (DEGs) in donor- (d) and host-derived (e) MPP Ly cells isolated from aged and young recipients (unpaired comparisons). The red and blue points represent the up- and down-regulated genes in cells isolated from aged recipients, respectively. (**f**) Venn diagrams displaying the overlap of DEGs between donor- and host-derived MPP Ly from young and aged recipients. (**g-h**) Volcano plots of DEGs between donor- and host-derived cells isolated from aged (g) and young (h) recipients (paired comparisons). The red and blue points represent the up- and down-regulated genes, respectively. (**f**) Radar plot displaying lineage affiliations associated with DEG identified in paired comparisons (g-h). In (c) and (e), colors denote the following groups: red, aged host cells; blue, young host; grey, young donor cells from aged hosts; dark grey, young donor cells from young hosts. In (d-e) and (g-h), DEGs were determined using an adjusted p-value threshold of < 0.1, with the Benjamini-Hochberg method applied to correct for multiple comparisons. Source data are provided as a Source data file.

Supplementary Fig. 5

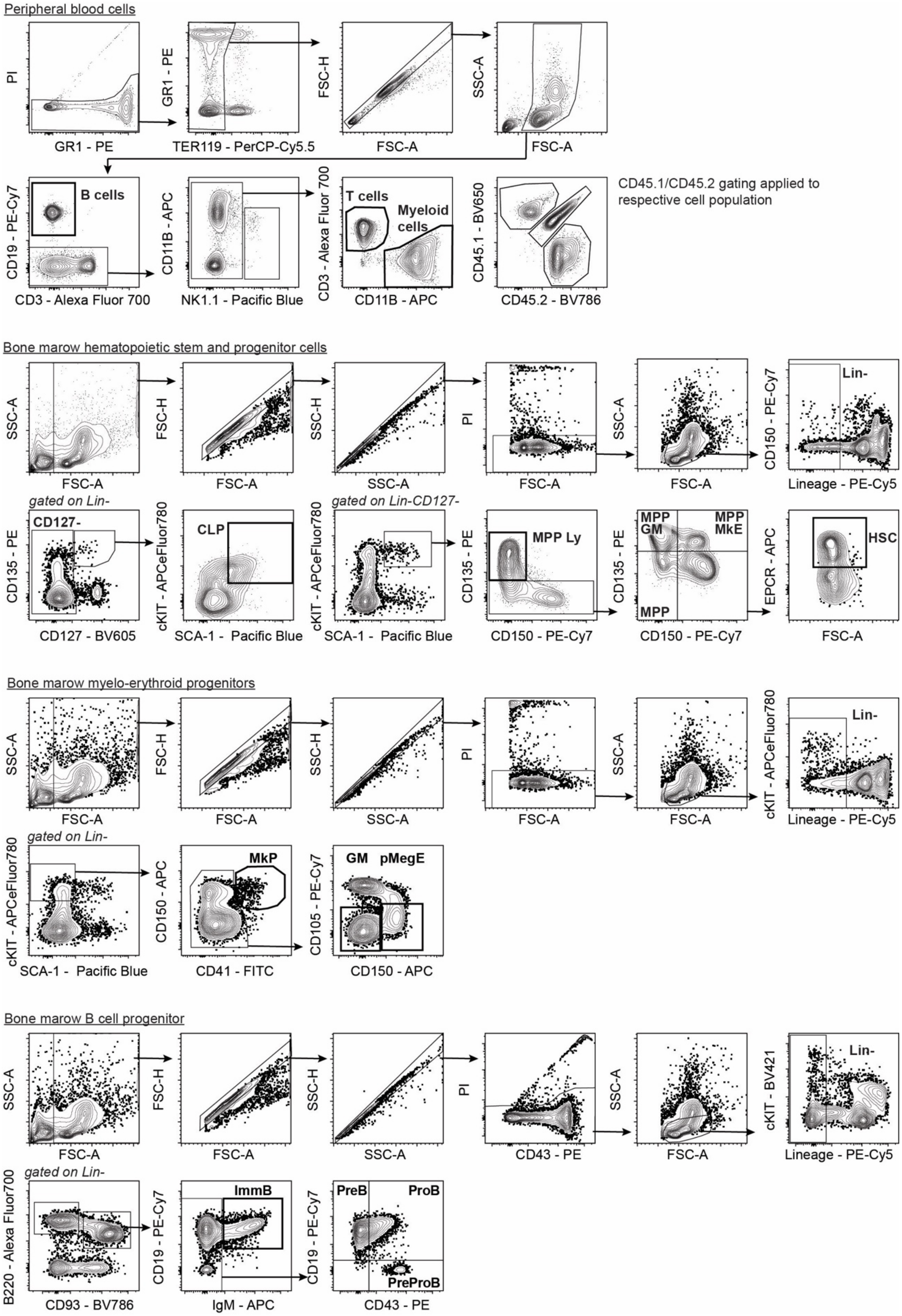

Supplementary Figure 5. Representative flow cytometric profiles of peripheral blood and bone marrow hematopoietic stem and progenitor cells. The flow cytometric plots display the gating strategies used to analyze peripheral blood and bone marrow cell subsets in this study.

Supplementary Fig. 6

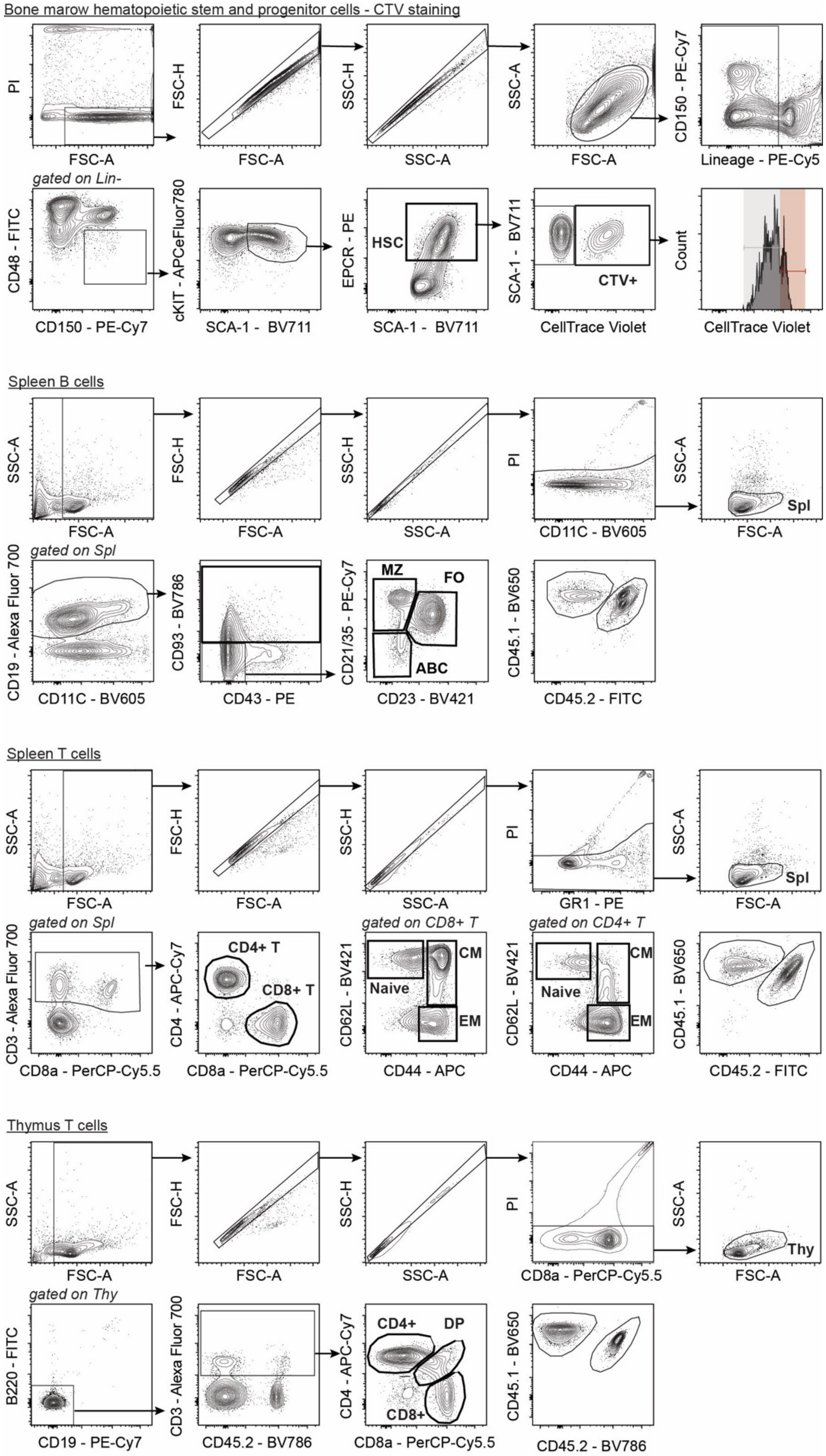

Supplementary Figure 6. Representative flow cytometric profiles of hematopoietic cells in the bone marrow, spleen, and thymus. The flow cytometric plots display the gating strategies used to analyze HSCs in the bone marrow, mature B and T cell subsets in the spleen, and T cells in the thymus.

Supplementary Table 1

Figure	Recipient mice	Recipient age at transplantation	Conditioning	Donor mice	Donor cells (NO. cells injected/recipient)
1F-G	Young and aged: C57BL/6-CD45.2	Young: 2-3 months<br>Aged: 16 months	CD45-SAP (3 mg/kg, day 0)	- *Fgd5*$^{CreERT2/+}$; *Rosa26*$^{LSL\text{-}tdTomato/+}$<br>- C57BL/6-CD45.1	- 500 tdTomato+ HSCs from young *Fgd5*$^{CreERT2/+}$; *Rosa26*$^{LSL\text{-}tdTomato/+}$ mice<br>- 500,000 BM cells from C57BL/6-CD45.1 mice
S1E	Aged: C57BL/6-CD45.2	16-19 months	Group 1: CD45-SAP (3 mg/kg, day 0)<br>Group 2: CD45-SAP (3 mg/kg, day 0) + CD8-SAP (0.5 mg/kg, day 6)<br>Group 3: CD45-SAP (3 mg/kg, day 0) + CD4-SAP (0.5 mg/kg, day 5)<br>Group 4: CD45-SAP (3 mg/kg, day 0) + anti-B220/CD22/CD19 (150 µg/mouse, day 3) + anti-rat kappa light chain (150 µg/mouse, day 5)	- *Fgd5*$^{CreERT2/+}$; *Rosa26*$^{LSL\text{-}tdTomato/+}$<br>- C57BL/6-CD45.1	- 500 tdTomato+ HSCs from young *Fgd5*$^{CreERT2/+}$; *Rosa26*$^{LSL\text{-}tdTomato/+}$ mice<br>- 500,000 BM cells from C57BL/6-CD45.1 mice
S1G	Young and aged: C57BL/6-CD45.2	Young: 2-3 months<br>Aged: 16 months	CD45-SAP (3 mg/kg, day 0) + TBI (200 cGy, day 7)	- *Fgd5*$^{CreERT2/+}$; *Rosa26*$^{LSL\text{-}tdTomato/+}$<br>- C57BL/6-CD45.1	- 500 tdTomato+ HSCs from young *Fgd5*$^{CreERT2/+}$; *Rosa26*$^{LSL\text{-}tdTomato/+}$ mice<br>- 500,000 BM cells from C57BL/6-CD45.1 mice
2B	Young: C57BL/6-CD45.1/CD45.2	3-5 months	Unconditioned	C57BL/6-CD45.1 and C57BL/6-CD45.2	Group 1: EE100 HSCs - total culture<br>Group 2: EE500 HSCs - Lin-EPCR+ fraction - single bolus<br>Group 3: EE100 HSCs - Lin-EPCR+ fraction - 5 weekly injections
2D-E	Young: C57BL/6-CD45.2	2-3 months	Group 1: CD45-SAP (3 mg/kg, day 0)<br>Group 2: TBI (950 cGy, 4 hours before transplantation)	C57BL/6-CD45.1	EE100 HSCs - total culture<br>For TBI-treated mice, cultured cells were co-transplanted with 500,000 BM cells from C57BL/6-CD45.2 mice
2G-H	Young: C57BL/6-CD45.1/CD45.2	2-4 months	TBI (950 cGy, 4 hours before transplantation)	Primary recipients from 2D-E	Group 1 (non-competitive) : 3x10^{6} BM cells from CD45-SAP-treated primary recipeints<br>Group 2 and 3 (competitive) : 3x10^{6} BM cells from CD45-SAP and TBI-treated primary recipeints mixed with 3x106 BM cells from C57BL/6-CD45.1x45.2 mice
3B-D	Young: C57BL/6-CD45.2 and C57BL/6-CD45.1/CD45.2<br>Aged: C57BL/6-CD45.2	Young: 2-4 months<br>Aged: 16 months	Group 1: Unconditioned<br>Group 2: CD45-SAP (3 mg/kg, day 0)	C57BL/6-CD45.1 or C57BL/6-CD45.2	- EE100 HSCs labeled with CTV dye<br>- 2x106 CD4-enriched splenocytes labeled with CTV dye
3F	Young: C57BL/6-CD45.1 and C57BL/6-CD45.2	2-3 months	TBI (950 cGy, 4 hours before transplantation)	Primary recipients from 3B-D	- 10 CTV^{high} HSCs from young and aged primary hosts<br>- 500,000 BM from C57BL/6-CD45.1 or C57BL/6-CD45.2 mice
4B-D	Aged: C57BL/6-CD45.2 and C57BL/6-CD45.1/CD45.2	Aged: 16-17 months	Group1: CD45-SAP (3 mg/kg, day 0)<br>Group2: CD45-SAP (3 mg/kg, day 0) + G-CSF (125 µg/kg, 2x/day, day 6-7) + AMD3100 (5 mg/kg, day 8)	C57BL/6-CD45.1	EE100 HSCs - total culture
4E-I	Young:C57BL/6-CD45.1/CD45.2<br>Aged: C57BL/6-CD45.2 and C57BL/6-CD45.1/CD45.2	Young: 2-3 months<br>Aged: 16-17 months	CD45-SAP (3 mg/kg, day 0) + G-CSF (125 µg/kg, 2x/day, day 6-7) + AMD3100 (5 mg/kg, day 8) (aged mice from Fig. 4B-D)<br>or G-CSF (125 µg/kg, 2x/day, day 0-1) + AMD3100 (5 mg/kg, day 2) + CD45-SAP (60 µg/mouse, 20 weeks after transplantation)	C57BL/6-CD45.1	EE100 HSCs - total culture
5B-D	NHD13^{tg} and WT littermates	2 months	CD45-SAP (3 mg/kg, day 0)	C57BL/6-CD45.1	10x10^{6} BM cells

Supplementary Table 1. Detailed information on transplantation experiments. Related to Fig. 1-5.

Summary of experimental design for transplantation experiments performed in this study. For experiments involving CD45-SAP treatment (with or without co-treatment with other conditioning agents), CD45-SAP was administered on day 0 and transplantation was performed on day 8. For experiments with CD45-SAP and G-CSF/AMD3100 co-treatment, transplantation was performed 1 hour after AMD3100 injection. See also Material and Methods. BM, unfractionated whole bone marrow; CTV, Cell Trace Violet; EE100 or EE500, cultured cells equivalent to expansion of 100 or 500 HSCs; TBI, total body irradiation; WT, wild-type.

Table S2. List of antibodies used in this study.

Antibody	Clone	Source	Cat. no.	Dilution
CD105 - PE/Cy7	MJ7/18	Biolegend	120410	1:200
CD11b - APC	M1/70	Sony Biotechnology	1106060	1:800
CD11c - Biotin	N418	Sony Biotechnology	1186520	1:200
CD16/32 - unconjugated	2.4G2	BioXCell	BE0307	1:100
CD16/32 - Alexa Fluor 700	93	eBioscience	56-0161-82	1:100
CD117 (cKIT) - APC	2B8	Sony Biotechnology	1129060	1:200
CD117 (cKIT) - APC/eFluor 780	2B8	eBioscience	47-1171-82	1:100
CD117 (cKIT) - Brilliant Violet 421	2B8	Sony Biotechnology	1129135	1:100
CD127 (IL7Ra) - Biotin	A7R34	Sony Biotechnology	1275030	1:200
CD135 (FLT3) - PE	A2F10	Sony Biotechnology	1276530	1:100
CD150 - PE/Cy7	TC150-12F12.2	Sony Biotechnology	1179570	1:200
CD150 - APC	TC150-12F12.2	Sony Biotechnology	1179550	1:200
CD19 - PE/Cy7	6D5	Sony Biotechnology	1177600	1:200
CD19 - Alexa Fluor 700	6D5	Sony Biotechnology	1177640	1:200
CD201 - PE	RCR-16	Sony Biotechnology	1307520	1:200
CD201 - APC	eBio1560	eBioscience	17-2012-82	1:200
CD21/35 - PE/Cy7	7E9	Biolegend	123420	1:200
CD23 - Pacific Blue	B3B4	Sony Biotechnology	1108080	1:200
CD3 - Alexa Fluor 700	17A1	Sony Biotechnology	1101080	1:200
CD3 - Biotin	17A1	Sony Biotechnology	1101220	1:200
CD3e - PE/Cy5	145-2C11	Sony Biotechnology	1101550	1:200
CD4 - APC/Cy7	GK1.5	BD Bioscience	A15384	1:200
CD4 - Biotin	GK1.5	Sony Biotechnology	1102020	1:200
CD41 - FITC	MWReg30	Sony Biotechnology	1269520	1:200
CD43 - PE	S7	BD Bioscience	553271	1:100
CD44 - APC	IM7	BD Bioscience	559250	1:200
CD45.1 - Alexa Fluor 700	A20	Sony Biotechnology	1153620	1:200
CD45.1 - Brilliant Violet 650	A20	Sony Biotechnology	1153680	1:100
CD45.2 - Biotin	104	Sony Biotechnology	109804	
CD45.2 - Brilliant Violet 785	104	Sony Biotechnology	1149195	1:100
CD45.2 - FITC	104	Sony Biotechnology	1149030	1:200
CD45R/B220 - Biotin	RA3-6B2	Sony Biotechnology	1116020	1:200
CD45R/B220 - Alexa Fluor 700	RA3-6B2	Sony Biotechnology	1116160	1:200
CD45R/B220 - PE/Cy5	RA3-6B2	Sony Biotechnology	1116050	1:200
CD45R/B220 - FITC	RA3-6B2	Sony Biotechnology	1116030	1:200
CD48 - FITC	HM 48-1	Sony Biotechnology	1117020	1:200
CD48 - Alexa Fluor 700	HM 48-1	Sony Biotechnology	1117130	1:200

CD62L - Brilliant Violet 421	MEL-14	Sony Biotechnology	1122175	1:200
CD8a - Biotin	53-6.7	Sony Biotechnology	1103520	1:200
CD8a - PerCP/Cy5.5	53-6.7	Sony Biotechnology	1103670	1:200
CD93 - Brilliant Violet 785	AA4.1	BD Bioscience	740941	1:100
Fcer1a - FITC	MAR-1	Sony Biotechnology	1271530	1:200
IgM - APC	RMM-2	Biolegend	406509	1:200
NK1.1 - Biotin	PK136	BD Bioscience	553163	1:200
NK1.1 - Pacific Blue	PK136	Sony Biotechnology	1143610	1:200
NK1.1 - PE/Cy5	PK136	Sony Biotechnology	1143580	1:200
Ly6A/E (SCA-1) - Pacific Blue	E13-161.7	Biolegend	122520	1:200
Ly6A/E (SCA-1) - BV711	E13-161.7	Sony Biotechnology	1140655	1:200
Ly6G/Ly6C (Gr1) - PE	RB6-8C5	Sony Biotechnology	1142040	1:400
Ly6G/Ly6C (Gr1) - Biotin	RB6-8C5	Sony Biotechnology	1142020	1:400
Ly6G/Ly6C (Gr1) – PE/Cy5	RB6-8C5	Sony Biotechnology	1142050	1:400
TER119 - PerCP/Cy5.5	TER119	Sony Biotechnology	1181140	1:400
TER119 - Biotin	TER119	Sony Biotechnology	1181020	1:400
TER119 - PE/Cy5	TER119	Sony Biotechnology	1181050	1:400
Streptavidin - Brilliant Violet 605		Sony Biotechnology	2626145	1:400
