# Supplementary material for: A non-genotoxic stem cell therapy boosts lymphopoiesis and averts age-related blood diseases in mice
Source: Nat Commun. 2025 Jun 2;16:5129. doi: 10.1038/s41467-025-60464-3 (PMC12130210; doi:10.1038/s41467-025-60464-3)
Supplement: Supplementary file 2 — Reporting Summary [file 41467_2025_60464_MOESM2_ESM.pdf]

nature portfolio

Corresponding author(s): David Bryder

Last updated by author(s): Apr 25, 2025

Reporting Summary

Nature Portfolio wishes to improve the reproducibility of the work that we publish. This form provides structure for consistency and transparency in reporting. For further information on Nature Portfolio policies, see our Editorial Policies and the Editorial Policy Checklist.

Statistics

For all statistical analyses, confirm that the following items are present in the figure legend, table legend, main text, or Methods section.

n/a	Confirmed	
☐	☒	The exact sample size (*n*) for each experimental group/condition, given as a discrete number and unit of measurement
☐	☒	A statement on whether measurements were taken from distinct samples or whether the same sample was measured repeatedly
☐	☒	The statistical test(s) used AND whether they are one- or two-sided<br>*Only common tests should be described solely by name; describe more complex techniques in the Methods section.*
☐	☒	A description of all covariates tested
☐	☒	A description of any assumptions or corrections, such as tests of normality and adjustment for multiple comparisons
☐	☒	A full description of the statistical parameters including central tendency (e.g. means) or other basic estimates (e.g. regression coefficient) AND variation (e.g. standard deviation) or associated estimates of uncertainty (e.g. confidence intervals)
☐	☒	For null hypothesis testing, the test statistic (e.g. *F*, *t*, *r*) with confidence intervals, effect sizes, degrees of freedom and *P* value noted<br>*Give P values as exact values whenever suitable.*
☒	☐	For Bayesian analysis, information on the choice of priors and Markov chain Monte Carlo settings
☒	☐	For hierarchical and complex designs, identification of the appropriate level for tests and full reporting of outcomes
☒	☐	Estimates of effect sizes (e.g. Cohen's *d*, Pearson's *r*), indicating how they were calculated

Our web collection on statistics for biologists contains articles on many of the points above.

Software and code

Policy information about availability of computer code

Data collection	Flow cytometry data were collected using LSRFortessa, Fortessa-X20, FACSAria III and FACSSymphony S6 instruments (Becton Dickinson). RNA sequencing data were collected using NovaSeq X Plus (Illumina).
Data analysis	Flow cytometry data were analyzed using FlowJo v.10.8.1 (Treestar). For analysis of RNA-sequencing data, the FASTQ files were aligned to the GRCm38 reference genome using STARsolo v.2.7.10b software. Downstream analyses were performed using the following software: R v.4.2.1, DESeq2 v.1.36.0, ggplot2 v.3.4.2, dplyr v.1.1.2, Enrichr (https://maayanlab.cloud/Enrichr/), Venny v.2.0.2 (https://bioinfogp.cnb.csic.es/tools/venny/index2.0.2.html). Additional software using in this study include Microsoft Excel v.16.66.1 and GraphPad Prism v.9.5.1.

For manuscripts utilizing custom algorithms or software that are central to the research but not yet described in published literature, software must be made available to editors and reviewers. We strongly encourage code deposition in a community repository (e.g. GitHub). See the Nature Portfolio guidelines for submitting code & software for further information.

Data

Policy information about availability of data

All manuscripts must include a data availability statement. This statement should provide the following information, where applicable:

- Accession codes, unique identifiers, or web links for publicly available datasets
- A description of any restrictions on data availability
- For clinical datasets or third party data, please ensure that the statement adheres to our policy

Bulk RNA-sequencing data has been deposited in the GEO database under the accession number GSE267079. No original code was developed in this study. Source Data are provided with this paper.

Research involving human participants, their data, or biological material

Policy information about studies with human participants or human data. See also policy information about sex, gender (identity/presentation), and sexual orientation and race, ethnicity and racism.

Reporting on sex and gender	*Use the terms sex (biological attribute) and gender (shaped by social and cultural circumstances) carefully in order to avoid confusing both terms. Indicate if findings apply to only one sex or gender; describe whether sex and gender were considered in study design; whether sex and/or gender was determined based on self-reporting or assigned and methods used. Provide in the source data disaggregated sex and gender data, where this information has been collected, and if consent has been obtained for sharing of individual-level data; provide overall numbers in this Reporting Summary. Please state if this information has not been collected. Report sex- and gender-based analyses where performed, justify reasons for lack of sex- and gender-based analysis.*
Reporting on race, ethnicity, or other socially relevant groupings	*Please specify the socially constructed or socially relevant categorization variable(s) used in your manuscript and explain why they were used. Please note that such variables should not be used as proxies for other socially constructed/relevant variables (for example, race or ethnicity should not be used as a proxy for socioeconomic status). Provide clear definitions of the relevant terms used, how they were provided (by the participants/respondents, the researchers, or third parties), and the method(s) used to classify people into the different categories (e.g. self-report, census or administrative data, social media data, etc.) Please provide details about how you controlled for confounding variables in your analyses.*
Population characteristics	*Describe the covariate-relevant population characteristics of the human research participants (e.g. age, genotypic information, past and current diagnosis and treatment categories). If you filled out the behavioural & social sciences study design questions and have nothing to add here, write "See above."*
Recruitment	*Describe how participants were recruited. Outline any potential self-selection bias or other biases that may be present and how these are likely to impact results.*
Ethics oversight	*Identify the organization(s) that approved the study protocol.*

Note that full information on the approval of the study protocol must also be provided in the manuscript.

Field-specific reporting

Please select the one below that is the best fit for your research. If you are not sure, read the appropriate sections before making your selection.

☒ Life sciences ☐ Behavioural & social sciences ☐ Ecological, evolutionary & environmental sciences

For a reference copy of the document with all sections, see nature.com/documents/nr-reporting-summary-flat.pdf

Life sciences study design

All studies must disclose on these points even when the disclosure is negative.

Sample size	Sample sizes were not pre-determined using statistical methods but are consistent with those reported in previous studies (f.i. Norddahl et al. 2013, Wahlestedt et al. 2013, Konturek-Ciesla et al. 2023). For all experiments, the number of mice per group is indicated in the corresponding figure legends. For transplantation of cultured cells, the equivalent of total expansion of 100 initially purified cells was transplanted into each recipient. In CTV experiments, the mice were additionally co-transplanted with 2,000,000 CTV-labelled CD4+ spleen cells. For transplantation experiments presented in Figure 1 and Supplementary Figure 1, 500 freshly purified hematopoietic stem cells were transplanted into each recipient. When indicated, the mice were co-injected with 500,000 or 1,000,000 support whole bone marrow cells. For bulk RNA-sequencing, 200 cells were sorted per sample.
Data exclusions	Animals other than NHD13tg showing symptoms of disease or distress were excluded from the analysis. A diseased state was defined as a hunched posture, lethargy, rapid or progressive weight loss or sizeable abdominal enlargement, in agreement with our ethical permits. One aged animal was excluded from the endpoint analysis (Fig. 4) based on severe abdominal swelling. Exclusion criteria were predetermined.
Replication	Each transplantation experiment presented in Figures 2, 3, 5 and Supplementary Figure 2 were performed once with n = 2-12 mice/group. Transplantation experiments presented in Figures 1, 4 and Supplementary Figure 1 and 3 were repeated 2-3 times and no inconsistent results

were observed. The results were successfully replicated. Evaluation of CD45-SAP depletion efficiency presented in Figure 1 and Supplementary Figure 1 was performed once.

Randomization

For transplantation experiments, no randomization method was applied to allocate animals to the experimental groups. Animals (n= 3–5) in the same cage received the same treatment.

Blinding

Blinding of investigators was not feasible, as information about mouse weight was necessary to administer conditioning reagents at the correct dose. For young mice undergoing repetitive transplantation, details about the dose and genotype of transplanted cells were required. Investigators were also aware of the age of the mice (based on physical appearance), further preventing blinding.

Reporting for specific materials, systems and methods

We require information from authors about some types of materials, experimental systems and methods used in many studies. Here, indicate whether each material, system or method listed is relevant to your study. If you are not sure if a list item applies to your research, read the appropriate section before selecting a response.

Materials & experimental systems

n/a	Involved in the study	
☐	☒	Antibodies
☒	☐	Eukaryotic cell lines
☒	☐	Palaeontology and archaeology
☐	☒	Animals and other organisms
☒	☐	Clinical data
☒	☐	Dual use research of concern
☒	☐	Plants

Methods

n/a	Involved in the study	
☒	☐	ChIP-seq
☐	☒	Flow cytometry
☒	☐	MRI-based neuroimaging

Antibodies

Antibodies used

CD105 - PE-Cy7 (Biolegend, Cat#120410, clone MJ7/18), dilution 1:200
CD11b -APC (Sony Biotechnology, Cat# 1106060, clone M1/70), dilution 1:800
CD11C - Biotin (Sony Biotechnology, Cat# 1186520, clone N418), dilution 1:200
CD16/32 - unconjugated (BioXCell, Cat# BE0307, clone 2.4G2), dilution 1:100
CD16/32 - Alexa Fluor 700 (eBioscience, Cat# 56-0161-82, clone 93), dilution 1:100
CD117 - APC (Sony Biotechnology, Cat# 1129060, clone 2B8), dilution 1:200
CD117 - APCeFluor780 (eBioscience, Cat# 47-1171-82, clone 2B8), dilution 1:100
CD117 - BV421 (Sony Biotechnology, Cat# 1129135, clone 2B8), dilution 1:100
CD127 - Biotin (Sony Biotechnology, Cat# 1275030, clone A7R34), dilution 1:200
CD135 - PE (Sony Biotechnology, Cat# 1276530, clone A2F10), dilution 1:100
CD150 - PE/Cy7 (Sony Biotechnology, Cat# 1179570, clone TC15-12F12.2), dilution 1:200
CD150 - APC (Sony Biotechnology, Cat# 1179550, clone TC15-12F12.2), dilution 1:200
CD19 - PE/Cy7 (Sony Biotechnology, Cat# 1177600, clone 6D5), dilution 1:200
CD19 - Alexa Fluor 700 (Sony Biotechnology, Cat# 1177640, clone 6D5), dilution 1:200
CD201 - APC (eBioscience, Cat# 17-2012-82, clone eBio1560), dilution 1:200
CD201 - PE (Sony Biotechnology, Cat# 1307520, clone rcr-16), dilution 1:200
CD21/35 - PE/Cy7 (Biolegend, Cat#123420, clone 7E9), dilution: 1:200
CD23 - Pacific Blue (Sony Biotechnology, Cat# 1108080, clone B3B4), dilution 1:200
CD3 -Alexa Fluor 700 (Sony Biotechnology, Cat# 1101080, clone 17A1), dilution 1:200
CD3 - Biotin (Sony Biotechnology, Cat# 1101220, clone 17A1), dilution 1:200
CD3 - PE/Cy5 (Sony Biotechnology, Cat# 1101550, clone 145-2C11), dilution 1:200
CD4 -APC/Cy7 (BD Bioscience, Cat# A15384, clone GK1.5), dilution 1:200
CD4 - Biotin (Sony Biotechnology, Cat# 1102020, clone GK1.5), dilution 1:200
CD41 - FITC (Sony Biotechnology, Cat# 1269520, clone MWReg30), dilution 1:100
CD43 - PE (BD Bioscience, Cat#553271, clone S7), dilution 1:100
CD44 - APC (BD Bioscience, Cat#559250, clone IM7), dilution 1:200
CD45.1 - Brilliant Violet 650 (Sony Biotechnology, Cat# 1153680, clone A20), dilution 1:100
CD45.1 - Alexa Fluor 700 (Sony Biotechnology, Cat# 1153620, clone A20), dilution 1:200
CD45.2 - Brilliant Violet 785 (Sony Biotechnology, Cat# 1149195, clone 104), dilution 1:100
CD45.2 - Biotin (Biolegend, Cat# 109804, clone 104)
CD45.2 - FITC (Sony Biotechnology, Cat# 1149030, clone 104), dilution 1:200
CD45R/B220 - Biotin (Sony Biotechnology, Cat# 1116020, clone RA3-6B2), dilution 1:200
CD45R/B220 - PE/Cy5 (Sony Biotechnology, Cat# 1116050, clone RA3-6B2), dilution 1:200
CD45R/B220 - Alexa Fluor 700 (Sony Biotechnology, Cat# 1116160, clone RA3-6B2), dilution 1:200
CD45R/B220 - FITC (Sony Biotechnology, Cat# 1116030, clone RA3-6B2), dilution 1:200
CD48 - FITC (Sony Biotechnology, Cat# 1117020, clone HM48-1), dilution 1:200
CD48 - Alexa Fluor 700 (Sony Biotechnology, Cat# 1117130, clone HM48-1), dilution 1:200
CD62L - BV421 (Sony Biotechnology, Cat#1122175, clone MEL-14), dilution 1:200
CD8a - Biotin (Sony Biotechnology, Cat#1103520, clone 53-6.7), dilution 1:200
CD8a - PerCP/Cy5.5 (Sony Biotechnology, Cat#1103670, clone 53-6.7), dilution 1:200
CD93 - Brilliant Violet 786 (BD Bioscience, Cat#740941, clone AA4.1), dilution 1:100

FceRIa - FITC (Sony Biotechnology, Cat#1271530, clone MAR-1), dilution 1:200
IgM - APC (Biolegend, Cat# 406509, clone RMM-1), dilution 1:200
NK1.1 - Biotin (BD Bioscience, Cat# 553163, clone PK136), dilution 1:200
NK1.1 - PE/Cy5 (Sony Biotechnology, Cat# 1143580, clone PK136), dilution 1:200
NK1.1 - Pacific Blue (Sony Biotechnology, Cat# 1143610, clone PK136), dilution 1:200
Ly6A/E (Sca-1) - Pacific Blue (Biolegend, Cat# 122520, clone E13-161.7), dilution 1:200
Ly6A/E (Sca-1) - BV711 (Sony Biotechnology, Cat# 1140655, clone D7), dilution 1:200
Ly6G/Ly6C (Gr-1) - Biotin (Sony Biotechnology, Cat# 1142020, clone RB6-8C5), dilution 1:400
Ly6G/Ly6C (Gr-1) - PE (Sony Biotechnology, Cat# 1142040, clone RB6-8C5), dilution 1:400
Ly6G/Ly6C (Gr-1) - PE/Cy5 (Sony Biotechnology, Cat# 1142050, clone RB6-8C5), dilution 1:400
Streptavidin - Brilliant Violet 605 (Sony Biotechnology, Cat# 2626145), dilution 1:400
Ter119 - Biotin (Sony Biotechnology, Cat# 1181020, clone TER119), dilution 1:400
Ter119 - PE/Cy5 (Sony Biotechnology, Cat# 1181050, clone TER119), dilution 1:400
Ter119 - PerCP/Cy5.5 (Sony Biotechnology, Cat# 1181140, clone TER119), dilution 1:400

Validation

The manufacturer has validated the antibodies for the specified applications. Validation profiles and references are available through the provided links. All antibodies were tested in panels before experimental procedures to establish optimal dilutions.

CD105 – PE-Cy7; species reactivity: Mouse; tested application: FC, product citations: 30; https://www.biolegend.com/en-ie/products/pe-cyanine7-anti-mouse-cd105-antibody-4573
CD11b – APC; species reactivity: Mouse, Human; tested application: FC, product citations: 32; https://www.sonybiotechnology.com/se/catalog/product/view/id/360/s/apc-anti-mouse-human-cd11b/category/2/
CD11C – Biotin; species reactivity: Mouse; tested application: FC, product citations: 22; https://www.sonybiotechnology.com/se/catalog/product/view/id/1467/s/biotin-anti-mouse-cd11c/category/2/
CD16/32 – unconjugated; species reactivity: Mouse; tested application: Fc receptor blocking in FC, ICC/IF, in vivo, product citations: 246; https://bioxcell.com/invivomab-anti-mouse-cd16-cd32-be0307?queryID=9f24e893990288d199c1466be71a5eab&objectID=30927&indexName=bioxcell_live_default_products
CD16/32 – Alexa Fluor 700; species reactivity: Mouse; tested application: FC, ICC/IF, neutralization, product citations: 36; https://www.thermofisher.com/antibody/product/CD16-CD32-Antibody-clone-93-Monoclonal/56-0161-82
CD117 -APC; species reactivity: Mouse; tested application: FC; product citations: 7; https://www.sonybiotechnology.com/se/catalog/product/view/id/69/s/apc-anti-mouse-cd117-c-kit/category/2/
CD117 - APCeFluor780; species reactivity: Mouse, Human; tested application: FC, FN, IV; citation: 94; https://www.thermofisher.com/antibody/product/CD117-c-Kit-Antibody-clone-2B8-Monoclonal/47-1171-80
CD117 – BV421; species reactivity: Mouse; tested application: FC; citation: 4; https://www.sonybiotechnology.com/se/brilliant-violet-421tm-anti-mouse-cd117-c-kit
CD127 - Biotin; species reactivity: Mouse; tested application: FC; citation: 5; https://www.sonybiotechnology.com/se/catalog/product/view/id/5754/s/biotin-anti-mouse-cd127-il-7ra/category/2/
CD135 -PE; species reactivity: Mouse; tested application: FC, product citations: 3; https://www.sonybiotechnology.com/se/catalog/product/view/id/5609/s/pe-anti-mouse-cd135/category/2/
CD150 - PE/Cy7; species reactivity: Mouse; tested application: FC, product citations: 17; https://www.sonybiotechnology.com/se/catalog/product/view/id/2441/s/pe-cyanine7-anti-mouse-cd150-slam/category/2/
CD150 - APC; species reactivity: Mouse; tested application: FC, product citations: 17; https://www.sonybiotechnology.com/se/catalog/product/view/id/2290/s/apc-anti-mouse-cd150-slam/category/2/
CD19 - PE/Cy7; species reactivity: Mouse; tested application: FC, product citations: 13; https://www.sonybiotechnology.com/se/catalog/product/view/id/1593/s/pe-cyanine7-anti-mouse-cd19/category/2/
CD19 - Alexa Fluor 700; species reactivity: Mouse; tested application: FC, product citations: 13; https://www.sonybiotechnology.com/se/catalog/product/view/id/2863/s/alexa-fluorr-700-anti-mouse-cd19/category/2/
CD201 - APC; species reactivity: Mouse; tested application: FC, product citations: 14; https://www.thermofisher.com/antibody/product/CD201-EPCR-Antibody-clone-eBio1560-1560-Monoclonal/17-2012-82
CD201 - PE; species reactivity: Mouse; tested application: FC, product citations: 1; https://www.sonybiotechnology.com/se/catalog/product/view/id/6680/s/pe-anti-mouse-cd201-epcr/category/2/
CD21 – PE/Cy7; species reactivity: Mouse; tested application: FC, product citations: 6; https://www.biolegend.com/en-ie/products/pe-cyanine7-anti-mouse-cd21-cd35-cr2-cr1-antibody-6228
CD23 - Pacific Blue; species reactivity: Mouse; tested application: FC, product citations: 6; https://www.sonybiotechnology.com/se/catalog/product/view/id/5475/s/pacific-bluetm-anti-mouse-cd23/category/2/
CD3 -Alexa Fluor 700; species reactivity: Mouse; tested application: FC, product citations: 10; https://www.sonybiotechnology.com/se/catalog/product/view/id/2843/s/alexa-fluorr-700-anti-mouse-cd3/category/2/
CD3-Biotin; species reactivity: Mouse; tested application: FC, product citations: 10; https://www.sonybiotechnology.com/se/catalog/product/view/id/9453/s/biotin-anti-mouse-cd3/category/2/
CD3 - PE/Cy5; species reactivity: Mouse; tested application: FC, product citations: 32; https://www.sonybiotechnology.com/se/catalog/product/view/id/29/s/pe-cyanine5-anti-mouse-cd3e/category/2/
CD4 - APC/Cy7; species reactivity: Mouse; tested application: FC, product citations: 5; https://www.thermofisher.com/antibody/product/CD4-Antibody-clone-GK1-5-Monoclonal/A15384
CD4 - Biotin; species reactivity: Mouse; tested application: FC, ICC, product citations: 11; https://www.sonybiotechnology.com/se/catalog/product/view/id/264/s/biotin-anti-mouse-cd4/category/2/
CD41 - FITC; species reactivity: Mouse; tested application: FC; product citations: 7; https://www.sonybiotechnology.com/se/catalog/product/view/id/5260/s/fitc-anti-mouse-cd41/category/2/
CD43 - PE; species reactivity: Mouse; tested application: FC; product citations: 65; https://www.bdbiosciences.com/en-us/products/reagents/flow-cytometry-reagents/research-reagents/single-color-antibodies-ruo/pe-rat-anti-mouse-cd43.553271
CD44 - APC; species reactivity: Mouse; tested application: FC; product citations: 141; https://www.bdbiosciences.com/en-us/products/reagents/flow-cytometry-reagents/research-reagents/single-color-antibodies-ruo/apc-rat-anti-mouse-cd44.559250
CD45.1 - Brilliant Violet 650; species reactivity: Mouse; tested application: FC, product citations: 19; https://www.sonybiotechnology.com/se/catalog/product/view/id/8514/s/brilliant-violet-650tm-anti-mouse-cd45-1/category/2/
CD45.1 – Alexa Fluor 700; species reactivity: Mouse; tested application: FC, product citations: 19; https://

www.sonybiotechnology.com/se/catalog/product/view/id/2866/s/alexa-fluorr-700-anti-mouse-cd45-1/category/2/
CD45.2 - Brilliant Violet 785; species reactivity: Mouse; tested application: FC, product citations: 12; https://www.sonybiotechnology.com/se/brilliant-violet-785tm-anti-mouse-cd45-2
CD45.2 - Biotin; species reactivity: Mouse; tested application: FC, product citations: 14; https://www.biolegend.com/en-ie/products/biotin-anti-mouse-cd45-2-antibody-5
CD45.2 - FITC; species reactivity: Mouse; tested application: FC, product citations: 12; https://www.sonybiotechnology.com/se/catalog/product/view/id/9/s/fitc-anti-mouse-cd45-2/category/2/
CD45R/B220-Biotin; species reactivity: Mouse, Human; tested application: FC, product citations: 13; https://www.sonybiotechnology.com/se/catalog/product/view/id/457/s/biotin-anti-mouse-human-cd45r-b220/category/2/
CD45R/B220 - PE/Cy5; species reactivity: Mouse, Human; tested application: FC, product citations: 13; https://www.sonybiotechnology.com/se/catalog/product/view/id/463/s/pe-cyanine5-anti-mouse-human-cd45r-b220/category/2/
CD45R/B220 - Alexa Fluor700; species reactivity: Mouse, Human; tested application: FC, product citations: 13; https://www.sonybiotechnology.com/se/catalog/product/view/id/2891/s/alexa-fluorr-700-anti-mouse-human-cd45r-b220/category/2/
CD45R/B220 - FITC; species reactivity: Mouse, Human; tested application: FC, product citations: 13; https://www.sonybiotechnology.com/se/fitc-anti-mouse-human-cd45r-b220
CD48-FITC; species reactivity: Mouse; tested application: FC, product citations: 2; https://www.sonybiotechnology.com/se/catalog/product/view/id/308/s/fitc-anti-mouse-cd48/category/2/
CD48 - Alexa Fluor 700; species reactivity: Mouse; tested application: FC, product citations: 2; https://www.sonybiotechnology.com/se/catalog/product/view/id/6035/s/alexa-fluorr-700-anti-mouse-cd48/category/2/
CD62L - BV421; species reactivity: Mouse; tested application: FC, product citations: 13; https://www.sonybiotechnology.com/se/brilliant-violet-421tm-anti-mouse-cd62l
CD8a - Biotin; species reactivity: Mouse; tested application: FC, product citations: 28; https://www.sonybiotechnology.com/se/catalog/product/view/id/150/s/biotin-anti-mouse-cd8a/category/2/
CD8a - PerCP/Cy5.5; species reactivity: Mouse; tested application: FC, product citations: 28; https://www.sonybiotechnology.com/se/catalog/product/view/id/3858/s/percp-cyanine5-5-anti-mouse-cd8a/category/2/
CD93 - Brilliant Violet 786; species reactivity: Mouse; tested application: FC, product citations: 9; https://www.bdbiosciences.com/en-us/products/reagents/flow-cytometry-reagents/research-reagents/single-color-antibodies-ruo/bv786-rat-anti-mouse-cd93-early-b-lineage.740941
FceRIa - FITC; species reactivity: Mouse; tested application: FC, product citations: 3; https://www.sonybiotechnology.com/se/catalog/product/view/id/5330/s/fitc-anti-mouse-fceria/category/2/
IgM-APC; species reactivity: Mouse; tested application: FC, product citations: 32; https://www.biolegend.com/en-ie/products/apc-anti-mouse-igm-2335
NKl.1-Biotin; species reactivity: Mouse; tested application: FC, product citations: 38; https://www.bdbiosciences.com/en-us/products/reagents/flow-cytometry-reagents/research-reagents/single-color-antibodies-ruo/biotin-mouse-anti-mouse-nk-1-1.553163
NKl.1-PE/Cy5; species reactivity: Mouse; tested application: FC, product citations: 15; https://www.sonybiotechnology.com/se/catalog/product/view/id/2569/s/pe-cyanine5-anti-mouse-nk-1-1/category/2/
NKl.1 - Pacific Blue; species reactivity: Mouse; tested application: IF, product citations: 15;
https://www.sonybiotechnology.com/se/catalog/product/view/id/2574/s/pacific-bluetm-anti-mouse-nk-1-1/category/2/
Ly6A/E {SCA-1) - Pacific Blue; species reactivity: Mouse; tested application: FC, product citations: 35; https://www.biolegend.com/en-ie/products/pacific-blue-anti-mouse-ly-6a-e-sca-1-antibody-3901
Ly6A/E {SCA-1) – BV711; species reactivity: Mouse; tested application: FC, product citations: 15; https://www.sonybiotechnology.com/se/reagents/flow-cytometry-reagents/brilliant-violet-711tm-anti-mouse-ly-6a-e-sca-1
Ly6G/Ly6C (Gr-1)-Biotin; species reactivity: Mouse; tested application: FC, product citations: 32; https://www.sonybiotechnology.com/se/catalog/product/view/id/473/s/biotin-anti-mouse-ly-6g-ly-6c-gr-1/category/2/
Ly6G/Ly6C (Gr-1) - PE/Cy5; species reactivity: Mouse; tested application: FC, product citations: 32; https://www.sonybiotechnology.com/se/catalog/product/view/id/479/s/pe-cyanine5-anti-mouse-ly-6g-ly-6c-gr-1/category/2/
Ly6G/Ly6C (Gr-1) - PE; species reactivity: Mouse; tested application: FC, product citations: 32; https://www.sonybiotechnology.com/se/catalog/product/view/id/477/s/pe-anti-mouse-ly-6g-ly-6c-gr-1/category/2/
TER119-Biotin; species reactivity: Mouse; tested application: FC, product citations: 8; https://www.sonybiotechnology.com/se/catalog/product/view/id/1540/s/biotin-anti-mouse-ter-119-erythroid-cells/category/2/
TER119 - PE/Cy5; species reactivity: Mouse; tested application: FC, product citations: 7; https://www.sonybiotechnology.com/se/catalog/product/view/id/1546/s/pe-cyanine5-anti-mouse-ter-119-erythroid-cells/category/2/
TER119 - PerCP/Cy5.5; species reactivity: Mouse; tested application: FC, product citations: 8;
https://www.sonybiotechnology.com/se/catalog/product/view/id/3927/s/percp-cyanine5-5-anti-mouse-ter-119-erythroid-cells/category/2/

Animals and other research organisms

Policy information about studies involving animals; ARRIVE guidelines recommended for reporting animal research, and Sex and Gender in Research

Laboratory animals

Mus musculus, C57BL/6-CD45.1, females, generated in house, young (2-4 months)
Mus musculus, C57BL/6-CD45.2, females, Jackson Laboratory, Janvier Labs, Taconic Bioscience , young (2-4 months) and aged (16-20 months)
Mus musculus, C57BL/6-CD45.1/CD45.2, females, generated in house, young (2-4 months) and aged (16-20 months)
Mus musculus, NHD13tg, males and females, Jackson Laboratory, RRID: IMSR_JAX:010505, young (2-3 months old)
All mice were housed in environment-enriched conditions with 12-hour light-dark cycles and water and food provided ad libitum.

Wild animals

The study did not include wild animals.

Reporting on sex

For the experiment using mouse model of MDS, both female and male NHD13tg and wild-type littermate mice were assigned to three groups, with each group contained an equal number of animals of both sexes. The total number of males was 15, and the total number of females was 19. Information about sex in transplanted group is included is Source Data file. Other transplantation

	experiments included female mice. The decision to use only females in most transplantation experiments was based on practical considerations that males exhibit aggression and fighting, which could interfere with our longitudinal experiments.
Field-collected samples	The study did not involve samples collected from the field.
Ethics oversight	All experimental procedures were performed according to the protocol permits M186-15 and 16468/2020, approved by Malmö-Lund Animal Experimentation Ethics Committee (Malmö - Lunds djurförsöksetiska nämnd).

Note that full information on the approval of the study protocol must also be provided in the manuscript.

Plants

Seed stocks	*Report on the source of all seed stocks or other plant material used. If applicable, state the seed stock centre and catalogue number. If plant specimens were collected from the field, describe the collection location, date and sampling procedures.*
Novel plant genotypes	*Describe the methods by which all novel plant genotypes were produced. This includes those generated by transgenic approaches, gene editing, chemical/radiation-based mutagenesis and hybridization. For transgenic lines, describe the transformation method, the number of independent lines analyzed and the generation upon which experiments were performed. For gene-edited lines, describe the editor used, the endogenous sequence targeted for editing, the targeting guide RNA sequence (if applicable) and how the editor was applied.*
Authentication	*Describe any authentication procedures for each seed stock used or novel genotype generated. Describe any experiments used to assess the effect of a mutation and, where applicable, how potential secondary effects (e.g. second site T-DNA insertions, mosiacism, off-target gene editing) were examined.*

Flow Cytometry

Plots

Confirm that:

☒ The axis labels state the marker and fluorochrome used (e.g. CD4-FITC).

☒ The axis scales are clearly visible. Include numbers along axes only for bottom left plot of group (a 'group' is an analysis of identical markers).

☒ All plots are contour plots with outliers or pseudocolor plots.

☒ A numerical value for number of cells or percentage (with statistics) is provided.

Methodology

Sample preparation	Tissue isolation: Peripheral blood was collected from the tail vein into EDTA-coated tubes (Sarstedt) or 2% (v/v) FBS/PBS with heparin (Leo Pharma, 5000 IE/ml diluted 1:500). For BM cell isolation, mice were euthanized by cervical dislocation, and femurs, tibias and hip bones were collected from both hind legs. Bones were crushed in ice-cold 2% (v/v) FBS/PBS. For isolation of thymus and spleen cells, organs were dissociated using a plunger and 70 µm strainer in ice-cold 2% (v/v) FBS/PBS. Single-cell suspensions were centrifuged at 400g for 10 min and filtered through 70 µm cell strainers prior to sample processing.<br>Staining using fluorescently-labeled antibodies: Stainings that included Brilliant Violet-conjugated reagents were supplemented with 10% Brilliant Stain Plus Buffer (BD). The cells were stained in 2% (v/v) FBS/PBS for 30 min at 4ºC in the dark, unless otherwise stated. For PB analysis, erythrocytes were sedimented with 1% Dextran T500 (Sigma-Aldrich) at 37°C for 30 min. The remaining erythrocytes were then lysed with ammonium chloride solution (STEMCELL Technologies) for 3 min at room temperature. Cells were stained in 2% (v/v) FBS/PBS with 2 mM EDTA (Vwr) and antibodies against TER119, CD19, CD11b, Gr1, NK1.1 and CD3.<br>HSPC analysis was performed on whole BM or cKIT-enriched cells. For cKIT enrichment, BM cells were stained with anti-cKIT-APC or anti-cKIT-APCeFluor780 antibody, followed by incubation with anti-APC MicroBeads (1:20, Miltenyi Biotec) for 30 min. Magnetic separation was performed using LS or MS columns and a manual separator, according to manufacturer's instructions (Miltenyi Biotec).<br>In all stainings, except for myelo-erythroid subsets, cells were incubated with Fc-block (1:50, BioXCell) for 15 min prior to antibody staining. For HSPC analysis, cells were stained with antibodies against lineage markers (B220, Gr1, TER119, NK1.1, CD3), SCA-1, cKIT, CD150, CD48, CD201 and in some experiments CD135 and CD127. Myelo-erythroid progenitors were identified using lineage markers (B220, Gr1, TER119, NK1.1, CD3), SCA-1, cKIT, CD150, CD105, CD16/32 and CD41. B cell progenitors were identified using lineage markers (Gr1, TER119, NK1.1, CD3), CD19, B220, IgM, CD93, CD43 and cKIT.<br>For thymocyte analysis, cells were stained with antibodies against CD19, B220, CD3, CD4 and CD8. For mature B and T cells, splenocytes were stained with antibodies against CD19, CD93, CD23, CD21/35, CD43, CD11b and CD11c for B cell lineage or Gr1, CD4, CD8, CD3, CD44 and CD62L for T cell lineage. In transplantation experiments, all staining panels included antibodies against CD45.1 and CD45.2 to monitor chimerism levels.<br>HSC isolation by FACS was performed on cKIT-enriched BM cells stained with the HSPC antibody cocktail described above. For isolation of cultured HSCs, cells were stained against lineage markers (Fcer1a, B220, Gr1, TER119, NK1.1, CD3) and CD201. Prior to analysis or sorting, cells were filtered and incubated with propidium iodide (1:1000, Invitrogen) to exclude dead cells.
Instrument	LSRFortessa, Fortessa-X20, FACSAria III and FACSSymphony S6 instruments (Becton Dickinson).
Software	BD Diva software v9.0 (BD Bioscience)<br>FlowJo 10 software vl0.5.3 (Treestar)

Cell population abundance	For transplantation of purified HSCs, 500 cells were sorted per sample. For HSC sorting for ex vivo expansion, 100 cells were sorted per well. For bylk RNA sequencing, 200 MPP Ly were sorted per sample. Sort purity was always determined by re-analysis of sorted cells.
Gating strategy	For cell analysis and sorting, single cells were gated using FSC-A/FSC-H and live nucleated cells were gated based on SSC-A/ FSC-A and Pl staining. Subsequently. different combination of surface markers were used to discriminate populations of interest in the bone marrow, spleen and thymus. For peripheral blood analysis, single cells were gated using FSC-A/FSC-H and live nucleated cells were gated based on SSC-A/ FSC-A, Pl and TER119 staining. Cells were subsequently gated based on CD19, CD3, CD11B and NKl.1 expression. Donor- and competitor-derived cells were discriminated by gating CD45.1 + and CD45.2+ cells different cell subsets. Representative gating strategy for analysis and sorting experiments performed in this study is included in Supplementary figures 5 and 6.

☒ Tick this box to confirm that a figure exemplifying the gating strategy is provided in the Supplementary Information.
